# Laparoscopic Surgery for Pheochromocytoma in Hemodialysis Patients

**DOI:** 10.1155/2022/3060647

**Published:** 2022-07-21

**Authors:** Shuichi Tatarano, Akihiko Mitsuke, Takashi Sakaguchi, Ryosuke Matsushita, Satoru Inoguchi, Hirofumi Yoshino, Hiroaki Nishimura, Yasutoshi Yamada, Hideki Enokida

**Affiliations:** Department of Urology, Graduate School of Medical and Dental Sciences, Kagoshima University, Kagoshima, Japan

## Abstract

**Objectives:**

We analyzed the clinical outcomes of laparoscopic adrenalectomy for pheochromocytomas in hemodialysis compared with nonhemodialysis patients.

**Methods:**

Fifty-seven patients (7 hemodialysis and 50 nonhemodialysis) were included in the study. We analyzed the differences in clinical parameters and outcomes between the hemodialysis patient groups and nonhemodialysis patient groups as well as identified predictors for an intraoperative hypertensive spike.

**Results:**

The increasing intravascular volume before surgery in hemodialysis patients made perioperative hemodynamic management safer. No significant difference in clinical parameters between the two groups was observed except for the length of hospitalization that was significantly longer in the hemodialysis patients (9 vs. 6 days, *P*=0.005). An increase in systolic blood pressure at CO_2_ insufflation was an independent predictor of a hypertensive spike with a cutoff value of 22.5 mmHg (odds ratio 1.038, 95% confidence interval 1.012–1.078).

**Conclusion:**

Laparoscopic adrenalectomy for pheochromocytomas in hemodialysis was safe and feasible. An increase in systolic blood pressure at CO_2_ insufflation was a predictor of the intraoperative hypertensive spike. The research in this manuscript is not registered. This is a retrospective study.

## 1. Introduction

Pheochromocytoma is a rare endocrine disease, occurring in 0.1 to 0.6% of patients with hypertension [1, 2]. Sustained or paroxysmal hypertension is the most common sign of pheochromocytoma. Measurement of urinary and plasma catecholamines and catecholamine metabolites is typically used to diagnose pheochromocytoma. However, diagnosing a pheochromocytoma is difficult in hemodialysis (HD) patients because symptoms such as hypertension are seen commonly, while anuria results in no urine sample available for testing [3]. Furthermore, pheochromocytomas are rare in HD patients, and we found only 22 cases in the English literature. To date, no study has compared the perioperative outcomes of pheochromocytoma surgery between HD and nonhemodialysis (non-HD) patients. Here, we reported our preoperative hemodynamic management strategies and the outcome of laparoscopic adrenalectomy for a pheochromocytoma in HD patients. We also investigated the factors predicting an intraoperative hypertensive spike in all patients with pheochromocytoma.

## 2. Patients and Methods

### 2.1. Patients

Between May 2007 and December 2020, 57 patients were admitted to our hospital for laparoscopic surgery of pheochromocytoma and were included in this retrospective study.

### 2.2. Preoperative Management

Preoperative antihypertensive agents were prescribed for all patients, even those who were normotensive, to prevent unpredictable intraoperative hemodynamic instability. All the patients were prescribed doxazosin, an alpha-adrenergic receptor blockade, with an initial dosage of 2 mg per day increased to a maximum of 16 mg per day if necessary. In addition, the baseline dry weight of the HD patients was increased by 0.5–1.0 kg the day before surgery to increase intravascular volume.

### 2.3. Surgical Procedure

The laparoscopic surgery was performed using a transperitoneal approach according to the surgeon's preference. After induction of general anesthesia, the patient was placed in a full-plank position. The first 10 mm trocar was placed, and a 12 mmHg CO_2_ pneumoperitoneum was created. The other trocars were then placed at four finger widths below the costal margin. The pneumoperitoneum was maintained at 8–10 mmHg throughout the entire procedure. The adrenal vessels were dissected and either clipped with vessel clips or sealed, and the tumor specimen was removed using an entrapment bag.

Intraoperative hypertension was controlled by intravenous administration of a vasodilator. The surgeon also paused the surgical procedure and worked with the anesthesiologist to stabilize the hemodynamic status. After removal of the pheochromocytoma, hypotension was treated with fluid and intravenous vasopressor agents as needed.

### 2.4. Data Analysis

We retrospectively analyzed patient characteristics in the HD and non-HD patient groups. The following clinical parameters were compared in the two groups: age, gender, body mass index (BMI), affected side, tumor size, preoperative systolic blood pressure (sBP), plasma catecholamine levels, iodine-123-metaiodobenzylguanidine (^123^I-MIBG) scintigraphy, smoking history, and use of oral antihypertensive drugs.

We also analyzed the following operative outcomes to evaluate the safety of laparoscopic adrenalectomy: operative time, estimated blood loss (EBL), intraoperative maximum sBP, and length of hospital stay.

We defined a sudden elevation in intraoperative sBP > 180 mmHg as a hypertensive spike, emulating the criteria in our previous publication [4]. We used univariate and multivariate analyses to determine the factors that predicted a hypertensive spike. Receiver operating characteristic (ROC) curve analysis was used to determine the minimum cutoff value of each factor that predicted a hypertensive spike.

### 2.5. Statistical Analysis

EZR software (Saitama Medical Center, Jichi Medical University), based on R (The R Foundation for Statistical Computing, version 4.0.2) and R Commander (version 2.7-0), was used for the statistical analyses in the study [5]. The relationships between the two patient groups were analyzed using the Mann–Whitney *U* test, chi-square analysis, and Fisher's exact test. Univariate and multivariate logistic regression were used to determine the factors that predicted an intraoperative hypertensive spike. *P* values < 0.05 were considered to indicate statistical significance.

## 3. Results

Of the 57 patients with pheochromocytoma, seven were assigned to the HD patient group (Table 1). The median (range) for age was 61 (44–67) years, the duration of HD, 21 (5–34) years, and the dosage of preoperative doxazosin, 12 (2–16) mg per day. In Case 6, plasma catecholamine levels were within the normal range, although ^123^I-MIBG scintigraphy showed radionuclide accumulation in the tumor.

There were 50 patients in the non-HD group.  Table 2 shows a comparison of patient demographic and clinical parameters between the HD and non-HD groups. No significant difference was observed for any of the parameters in either group. As shown in Table 3, there were also no significant differences between the two groups for operative outcomes, including operative time, EBL, sBP elevation at CO_2_ insufflation, and maximum intraoperative sBP. The anesthesiologist used antihypertensive and antihypotensive medications during the surgery. Examples of such drugs include phentolamine mesylate, nicardipine hydrochloride, diltiazem hydrochloride, noradrenaline, and dopamine hydrochloride. Patients requiring treatment for hypertension during the surgery were 6 of 7 (86%) in the HD group and 41 of 50 (82%) in the non-HD group (*P*=>0.999). Patients requiring treatment for hypotension after the surgery were 1 of 7 (14%) in the HD group and 5 of 50 (10%) in the non-HD group (*P*=0.562). All the patients with postoperative hypotension recovered on postoperative day 1. However, we found that the length of hospital stay in the HD group was significantly longer than in the non-HD group (9 vs. 6 days, *P*=0.005). Intraoperative fluid balance was also significantly less than in the non-HD group (720 vs. 1950 mL, *P*=0.004) (Table 3). There were no intraoperative complications, and the postoperative course was uneventful in both groups.

**Figure 1:**
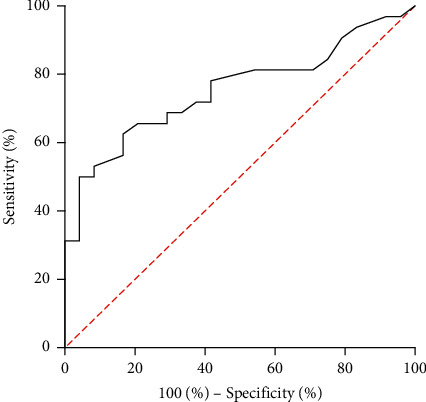
ROC curve analysis of systolic blood pressure elevation during pneumoperitoneum creation predicting the hypertensive spike. The sensitivity was 62.5% and specificity was 83.3% when systolic blood pressure increased > 22.5 mmHg from baseline levels.

The univariate and multivariate analyses to identify clinical factors that predicted a hypertensive spike are shown in Table 4. Of the significant predictors identified in the univariate analysis, sBP elevation at CO_2_ insufflation (odds ratio 1.038, 95% confidence interval 1.012–1.078, *P*=0.017) was the only independent predictive factor identified in the multivariate analysis. ROC curve analysis showed that an increase of 22.5 mmHg in sBP at CO_2_ insufflation had a sensitivity of 62.5% and a specificity of 83.3% (area under the ROC curve 0.754, *P*=0.001) (Figure 1). Four patients (57%) in the HD group and 28 (56%) in the non-HD group had a hypertensive spike. This difference between the two groups was not statistically significant. Of the patients who experienced a hypertensive spike, 4 (100%) HD patients and 16 (57%) non-HD patients had an increase in sBP at CO_2_ insufflation that exceeded the cutoff value used to predict a crisis (i.e., >22.5 mmHg).

## 4. Discussion

Pheochromocytoma is a rare catecholamine-secreting tumor that occurs in 0.1 to 0.6% of patients with hypertension [1, 2]. It is estimated that the annual incidence of pheochromocytoma is approximately 0.8 per 100,000 person-years [6]. The symptoms consist of episodic headaches, sweating, and tachycardia in approximately 50% of patients with pheochromocytoma, and when present, they are typically paroxysmal. Hypertension, the most common abnormality, occurs in more than 90% of patients and is paroxysmal in 25% to 50% of cases [7].

Pheochromocytoma in an HD patient is rare. To our knowledge, only 22 cases of pheochromocytoma in association with HD have been reported in the English literature [8–11]. The diagnosis of pheochromocytoma in HD patients can be challenging. Unfortunately, because of aneurin, only blood testing is available. Plasma catecholamine levels in HD patients is generally higher than those in normal controls, although a three-fold increase in plasma catecholamine should raise the suspicion of pheochromocytoma [3]. In imaging studies, computed tomography (CT) and magnetic resonance imaging (MRI) have similar sensitivity (93–100%) and specificity (50%). This low specificity is a major problem in both imaging techniques. In contrast, MIBG scintigraphy has superior specificity (95%–100%), although false-negative results have been reported in 10–15% of cases with pheochromocytoma [12, 13]. In our study, all seven cases had positive results for I^123^-MIBG scintigraphy, despite a false-negative result for MIBG scintigraphy having been reported in a previous case report of an HD patient [8]. These findings suggest that it is necessary to diagnose pheochromocytoma in HD patients based on the levels of blood catecholamine or catecholamine metabolites and imaging findings such as CT, MRI, and MIBG scintigraphy.

Once a pheochromocytoma is diagnosed, all patients should undergo resection of the tumor following appropriate medical preparation. Hemodynamic instability during the surgical procedure for a pheochromocytoma with inadequate or absent preoperative antihypertensive therapy or unrecognized hypovolemia contributes to a high mortality rate. Preoperative antihypertensive treatment is therefore recommended for patients with sustained or paroxysmal hypertension and also normotensive patients [14]. However, there is no evidence of the optimal endpoint for blood pressure. Retrospective studies suggest that the endpoint for blood pressure of less than 130/80 mmHg while seated and greater than 90 mmHg systolic while standing seems reasonable [15]. The perioperative management of HD patients is essentially the same as that used in non-HD patients [16]. However, preoperative hemodialysis may result in insufficient extracellular fluid volume, which in turn may lead to hypotension after the removal of the tumor. In some reports on the optimal adjustment of dry weight, a 1% to 3% increase in dry weight is recommended in HD patients [11, 17, 18]. Based on the findings of these reports, we approached surgery by increasing the dry weight preoperatively. Using this strategy, we encountered no significant problems with intraoperative hemodynamics in our cases.

Minimally invasive adrenalectomy is a safe procedure for the resection of pheochromocytoma [19, 20]. To date, there are only a small number of reports on laparoscopic surgery for pheochromocytoma in HD patients, and to the best of our knowledge, this is the first report to show that laparoscopic surgery for pheochromocytoma was comparable in HD and non-HD patients. However, the length of hospital stay in the HD group was longer because of the perioperative HD schedule.

Previous retrospective studies have reported that the factors that predicted severe intraoperative hypertension included preoperative preparation, catecholamine levels, intraoperative management, tumor characteristics, and the surgical approach [15, 21, 22]. Occasionally, we experience an increase in sBP shortly after the CO_2_ insufflation. This phenomenon may be because of pneumoperitoneum pressure on the tumor. However, there are no reports on the correlation between pneumoperitoneum and hemodynamics in laparoscopic pheochromocytoma surgery. Only one prospective study identified tumor size as a factor that predicted an intraoperative hypertensive spike (i.e., sBP > 180 mmHg) [4]. In our univariate analysis of the predictive factor for an intraoperative hypertensive spike, catecholamines and preoperative sBP were included as variables related to increased blood pressure. In addition, tumor size, operative time, and postpneumoperitoneum blood pressure increase were included in the variables considering the effects of the surgical procedure. Tumor size was not a significant predictive factor, whereas an increase in baseline sBP > 22.5 mmHg at CO_2_ insufflation was a strong predictor. In particular, all four dialysis patients with an intraoperative hypertensive spike had a higher increase in sBP than the cutoff value. Therefore, if sBP is elevated significantly during CO_2_ insufflation, it is necessary to pay close attention to the possibility of an intraoperative hypertension spike during the surgical procedure.

This study had several limitations as it was a retrospective study carried out at a single center and included only a small number of cases due to pheochromocytoma being a rare disease. In addition, the effects of differences in the surgical procedures could not be ruled out.

## 5. Conclusion

Laparoscopic adrenalectomy for pheochromocytoma is feasible in both HD and non-HD patients. Increasing the intravascular volume before surgery in HD patients appeared to make perioperative management safer. However, further study is required to determine an adequate level for increasing preoperative dry weight. Our data indicate that an increase in sBP at CO_2_ insufflation may be a predictive factor for a hypertensive spike during laparoscopic surgery for pheochromocytoma.

## Figures and Tables

**Table 1:** Characteristics of the hemodialysis patient with pheochromocytoma.

Case	Age	Sex	HD duration (yr)	Tumor size (mm)	Doxazosin (mg/day)	E (pg/mL)	NE (pg/mL)	DA (pg/mL)	MIBG uptake
1	59	Male	11	29	2	385	906	17	yes
2	61	Female	34	25	2	50	610	40	yes
3	61	Female	30	40	10	273	525	76	yes
4	48	Female	26	22	12	257	1328	28	yes
5	44	Female	5	47	16	395	2818	24	yes
6	64	Male	21	60	16	64	233	13	yes
7	67	Female	17	26	16	80	599	18	yes

HD, hemodialysis; E, epinephrine; NE, norepinephrine; DA, dopamine; MIBG, metaiodobenzylguanidine.

**Table 2:** Comparison of clinical parameters in the HD and non-HD patient groups.

	HD	Non-HD	*P* value
Number	7	50	
Age	61 (44–67)	56 (24–86)	0.948
Sex (male/female)	2/5	28/22	0.173
BMI (kg/m^2^)	22.2 (19.3–28.2)	22.3 (16.2–28.7)	0.762
Side (right/left)	3/4	25/25	0.723
Tumor size (mm)	29 (22–60)	40 (15–95)	0.282
Preoperative Sbp	120 (114–208)	129 (87–170)	0.597
Epinephrine (pg/mL)	257 (50–395)	148 (15–3221)	0.563
Norepinephrine (pg/mL)	610 (233–2818)	1454 (198–8112)	0.131
Dopamine (pg/mL)	24 (13–76)	18 (5–216)	0.190
MIBG uptake (yes/no)	7/0	48/1	>0.999
Smoking (yes/no)	1/6	22/28	0.223
Antihypertensive drug (yes/no)	6/1	25/25	0.112

HD, hemodialysis; BMI, body mass index; sBP, systolic blood pressure; MIBG, metaiodobenzylguanidine.

**Table 3:** Operative outcomes in the HD and non-HD patient groups.

	HD	Non-HD	*P* value
Operative time (min)	213 (113–321)	159 (32–363)	0.200
EBL (mL)	50 (20–320)	20 (3–880)	0.138
Intraoperative sBP max (mmHg)	195 (131–288)	199 (114–300)	0.804
sBP elevation at CO_2_ insufflation (mmHg)	35 (0–177)	19 (0–136)	0.640
Time to sBP elevation at CO_2_ insufflation (min)	5 (3–20)	6 (1–19)	0.813
Hypertensive spike number (%)	4 of 7 (57%)	28 of 50 (56%)	>0.999
Exceeded the cutoff value of 22.5 mmHg (%)	4 of 4 (100%)	16 of 28 (57%)	0.271
Intraoperative fluid balance (mL)	720 (708–1315)	1950 (1540–2425)	0.004
Required treatment for hypertension during surgery	6 of 7 (86%)	41 of 50 (82%)	>0.999
Required treatment for hypotension after surgery	1 of 7 (14%)	5 of 50 (10%)	0.562
Hospital stays (day)	9 (7–11)	6 (3–18)	0.005

HD, hemodialysis; EBL, estimated blood loss; sBP, systolic blood pressure.

**Table 4:** Univariate and multivariate analyses of clinical factors for predicting a hypertensive spike in all the patients.

	Univariate			Multivariate		
	OR	95% CI	*P* value	OR	95% CI	*P* value
Tumor size	1.018	0.991–1.050	0.215	—	—	—
Epinephrine	1.002	1.000–1.004	0.855	—	—	—
Norepinephrine	1.000	1.000–1.001	0.086	0.999	0.995–1.001	0.468
Epinephrine + norepinephrine	1.000	1.000–1.001	0.035	1.002	0.999–1.005	0.366
Preoperative sBP	0.996	0.969–1.023	0.752	—	—	—
Operative time	1.005	0.997–1.015	0.219	—	—	—
sBP elevation at CO_2_ insufflation	1.044	1.018–1.082	0.005	1.038	1.012–1.078	0.017

OR, odds ratio; CI, confidence interval; sBP, systolic blood pressure.

## Data Availability

No data were used to support this study.
